# Antibiotics Alter the Expression of Genes Related to Behavioral Development in Honey Bees (Hymenoptera: Apidae)

**DOI:** 10.1093/jisesa/ieac017

**Published:** 2022-04-07

**Authors:** Yarira Ortiz-Alvarado, Tugrul Giray

**Affiliations:** Department of Biology, University of Puerto Rico, Rio Piedras, SJ 00925, Puerto Rico

**Keywords:** honey bee, antibiotics, behavioral development, gene expression, insulin pathway

## Abstract

Honey bees, as many species of social insects, display a division of labor among colony members based on behavioral specializations related to age. Adult worker honey bees perform a series of tasks in the hive when they are young (such as brood care or nursing) and at ca. 2–3 wk of age, shift to foraging for nectar and pollen outside the hive. The transition to foraging involves changes in metabolism and neuroendocrine activities. These changes are associated with a suite of developmental genes. It was recently demonstrated that antibiotics influence behavioral development by accelerating or delaying the onset of foraging depending on timing of antibiotic exposure. To understand the mechanisms of these changes, we conducted a study on the effects of antibiotics on expression of candidate genes known to regulate behavioral development. We demonstrate a delay in the typical changes in gene expression over the lifetime of the individuals that were exposed to antibiotics during immature stage and adulthood. Additionally, we show an acceleration in the typical changes in gene expression on individuals that were expose to antibiotics only during immature stage. These results show that timing of antibiotic exposure alter the typical regulation of behavioral development by metabolic and neuroendocrine processes.

Antibiotics in apiculture are used to treat or prevent infections and to improve honey production (van Veen et al. 2014). The most common antibiotic used is oxytetracycline. Oxytetracycline is a broad-spectrum antibiotic used to treat honey bee (*Apis mellifera*; Linnaeus 1758) colonies against bacterial infections of American foulbrood (AFB; Elzen et al. 2002, Tian et al. 2012, Rokop et al. 2015). Antibiotic treatments in honey bee colonies are associated with changes in microbiota composition (Raymann et al. 2018, Ortiz-Alvarado 2019) and reduction of honey bee life span (Raymann et al. 2017) as well as changes in lipid metabolism and behavioral development (Ortiz-Alvarado et al. 2020).

In honey bees, young workers usually perform tasks inside the colony, while older workers perform tasks outside the colony (Seeley 1982, Winston 1991). Young bees (1–3 d old) perform cleaning tasks, middle-aged nurse bees (7–10 d) feed larvae and older bees (14+ d) perform foraging tasks. This change of task by age is known as behavioral development (Giray and Robinson 1994). While age is a factor, several others can influence the rate of behavioral development such as starvation (Schulz et al. 1998), nutritional state (Toth and Robinson 2005, Toth et al. 2005), and gene expression (Whitfield et al. 2003, Nilsen et al. 2011, Shi et al. 2012). As a result, workers of the same age can be observed performing different tasks or workers of different ages performing same tasks (Huang and Robinson 1992, Giray and Robinson 1996, Giray et al. 1999), demonstrating plasticity in behavioral development.

Recently, it was demonstrated that applying antibiotics to the honey bee hive also promotes differences in behavioral development (Ortiz-Alvarado et al. 2020). Bees treated with antibiotics for a prolonged period, covering immature and adult development stages, expressed a delayed behavioral development with fewer than 10% of the individuals foraging at 4 wk of age. In contrast, bees that were treated with antibiotics only during immature development, larval to pupal stage, displayed an accelerated behavioral development as adults. The effects could be a direct consequence of toxicity to the bee (*toxic effect hypothesis*) as with other chemicals (Tavares et al. 2017, Tome et al. 2020). Alternately, antibiotics indirectly may alter signaling from microbiota shown to be associated with behavioral tasks (*regulatory effect hypothesis*) in honey bees (Martinson et al. 2012, Jones et al. 2018).

Honey bee nurses and foragers have different gene expression profiles, where genes that are upregulated in typical nurses are downregulated in typical foragers and vice versa in a tissue-specific manner (Whitfield et al. 2003, Doke 2017,Breshanan et al. 2022). Among the regulatory factors related to behavioral development are juvenile hormone (JH) typically high at the onset of foraging (Robinson 1987, Giray and Robinson 1996, Sullivan et al. 2000), vitellogenin (Vg) higher in nurses than foragers (Amdam et al. 2003, 2006), and components of the insulin–insulin signaling (IIS) metabolic pathway such as insulin-like peptides (ILPs), insulin receptor substrate (IRS), and target of rapamycin (TOR; Corona et al. 2007; Ament et al. 2008, 2011; Nilsen et al. 2011).

ILP’s function is homologous to insulin in mammals (Wang et al. 2011, Nilsen et al. 2011), mediating the metabolic regulatory network (Britton et al. 2002, Ament et al. 2011). In bees, ILP-1 is highly expressed in foragers, whereas ILP-2 is highly expressed in nurses (Ament et al. 2008). IRS controls signaling of IIS and target of rapamycin (TOR) regulates growth in response to nutrient status and both have lower expression levels in nurse bees compared with foragers (Ament et al. 2008, 2011; Nilsen et al. 2011).

In this study, we evaluated the effects of the antibiotic oxytetracycline by quantifying and comparing expression profiles of genes associated with development; IIS genes (IRS, ILP-1, ILP-2), TOR, Vg- and JH-related genes, in four different antibiotic treatment groups during different stages of development. Our main hypothesis, *regulatory effect hypothesis* states that antibiotics will influence the metabolic changes bees undergo during behavioral development through changes in expression of the complex of regulatory gene pathways. We expected bees related to the accelerated behavioral development phenotype to have higher expression levels of genes related to forager tasks at a chronologically earlier age. We also expected bees related to the delayed behavioral development phenotype to have higher expression levels of genes related to nursing tasks through chronologically later ages. *Toxic effect hypothesis* does not lead to a prediction of coordinated or bidirectional changes in regulator gene expression. Instead, harm may be reduced in individuals exposed for a shorter period of time; only adult or only immature exposure to antibiotics.

## Materials and Methods

### Antibiotic Treatment and Sample Collection

Samples in this study were collected concurrently with the behavioral and fat metabolism study of Ortiz-Alvarado et al. (2020). On the basis of the results of the behavioral study, we designed the gene expression study, bees were sampled at random from each treatment group. Four typical colonies were screened and assessed to be free of disease and paired based on colony composition and population to be randomly assigned a treatment; Control: treated with powdered sugar or Antibiotic: treated with the antibiotic Oxytetracycline (Terra-Pro; Mann Lake Hackensack, MN) following the recommended dose from the manufacturer of 200 mg of oxytetracycline weekly over a course of three weeks. After initial antibiotic treatment, brood frames at 3 wk were collected from each pair of colonies and placed in an incubator for a period of 24 h at 35°C, when bees emerged we cross-fostered them as described in Ortiz-Alvarado et al. (2020). Briefly, approximately 200 newly emerged bees were collected from each colony. Half of the bees collected from one colony were marked with a color on their thorax to identify colony of origin and treatment, and returned to their original colony. The other half was marked with a different color and introduced to the pair colony, this is done for all paired colonies. Each colony continued their initial treatment; control (powder sugar) or antibiotic. The cross-fostering design results in four different treatment groups: No exposure (−/−) bees raised in control colony, kept in control colony; developmental exposure (+/−) bees emerged from antibiotic colony, introduced into the control colony after emergence; adult exposure (−/+) bees emerged from control colony, introduced into the antibiotic colony after emergence; and prolonged exposure (+/+) bees raised in antibiotic colony and kept in antibiotic colony.

We collected five bees from each of the colonies at the ages of 1, 7, and 14 days old per treatment group. Since bees were newly emerged and thus not cross-fostered at 1 d of age, bees of day 1 were collected only from the −/− and +/+ groups. We had a total of 100 collected bees. Age of collection was selected to be paired with onset of behaviors as described by Seeley (1982) and Moore et al. (1998). Samples (whole body) were placed in a microtube with 1 ml of RNAlater-ICE reagent (Ambion Life Technologies) and stored at −80°C for RNA extraction.

### Gene Selection

To examine the genic effects of antibiotics during development among the different treatment groups, we chose targets related to development and metabolic pathways. JH serves as an indicator of behavioral developmental rate, and JH levels increase as the worker bee ages (Robinson et al. 1989, Huang and Robinson 1996, Jassim et al. 2000). Vg is related to lipid synthesis in honey bees, nutritional status, and nursing behavior (Trenczek et al. 1989, Nelson et al. 2007). IIS components (ILP-1, ILP-2, and IRS) are involved in the metabolic signaling pathways that drive stable lipid loss (Corona et al. 2007, Ament et al. 2011, Nilsen et al. 2011) and TOR regulates growth in response of nutrient status and is regulated by IIS and upstream Vg synthesis (Patel et al. 2007, Ament et al. 2008).

Primers for gene sequences related to JH levels were designed. Since JH is a sesquiterpenoid and is not amenable to be measured through direct gene expression, its levels were determined indirectly by measuring JH acid methyltransferase (JHamt). JHamt is an enzyme that converts JH acids, inactive precursor of JH, to active JH at the final stage of biosynthesis pathway in insects and acts as a regulator during insect metamorphosis (Shinoda and Itoyama 2003, Minakuchi et al. 2008, Niwa et al. 2008). Studies conducted by Bomtorin et al. (2014) determined that JHamt is higher in forager bees than in nurses. Sequence from JHamt *A. mellifera* (accession number; JQ858262.1) was used for primer design using the program Primer3 by NCBI (Ye et al. 2012). For primer design we used the criteria as explained in Ortiz-Alvarado and Rivera-Marchand (2020). In brief, primers consisted of a length of 20 nucleotides, a melting temperature that did not exceed two degrees of difference between each primer pair, homodimer, and heterodimer with 5′ end free and ΔG from −3 to positive. Designed primers were tested and optimized in a PCR reaction prior to quantitative PCR (qPCR) runs.

Primers for Vg, IRS, ILP-1, ILP-2, TOR, and reference genes were obtained from the literature from experiments related to behavioral development in honey bees (Corona et al. 2007; Scharlaken et al. 2008; Mutti et al. 2011). List of target genes, accession numbers, primer sequence, TM, and % of efficiency are shown in Table 1.

**Table 1.** Primer list. Honey bee development-associated gene primers used for qPCR analysis. Target and housekeeping genes, primer sequences are provided along with their accession numbers, TM used, and calculated primer efficiencies

Target gene	Gene description	GeneBank acc. no.	Primer sequence Fw and Rv	TM (°C)	% Efficiency
*JHamt*	Juvenile hormone acid methyltransferase	JQ858262.1	TTGGACATAGGTTGCGGACC AATCCTTTTCCTCCTGGCCG	57	97.24
*Vg*	Vitellogenin	NP_001011578	AGTTCCGACCGACGACG TTCCCTCCCACGGAGTCC	57	93.43
*IRS*	Insulin receptor substrate	XM_391985	TTTGCAGTCGTTGCTGGTA TAGCGGTAGTGGCACAGTTG	57	89.91
*TOR*	Target of rapamycin	XM_625127	AACAACTGTTGCTGACGGTG GTTGCAGTCCAGGCTTTTTG	56	92.71
*ILP1*	Insulin-like peptide 1	GB17332	CGATAGTCCTGGTCGGTTTG CAAGCTGAGCATTGCAC	55	98.03
*ILP2*	Insulin-like peptide 2	GB10174	TTCCAGAAATGGAGATGGATG TAGGAGCGCAACTCCTCTGT	52	99.66
*RPL32*	Ribosomal protein L32	NM_001011587	TGTGCTGAAATTGCTCATGG CGTAACCTTGCACTGGCATA	55	103.98
*GAPDH*	Glyceraldehyde 3-phosphate dehydrogenase	XM_393605	GATGCACCCATGTTTGTTTG TTTGCAGAAGGTGCATCAAC	53	101.78

### RNA Extraction and cDNA Synthesis

RNA was extracted from two tissues, brain and abdomen, using the Trizol (Invitrogen, Carlsbad, CA) total RNA isolation method. Heads and abdomens from individual bees stored previously in RNAlater-ICE reagent were separated and kept on dry ice until dissections were finished. Brains were dissected on dry ice; compound eyes, ocelli, hypopharyngeal glands, were removed during dissection. For abdomen the stinger apparatus was removed alongside the gut. The dissected brain and the remaining abdomen tissue were kept separately and each were mechanically homogenized. RNA samples were quantified in a Nanophotometer (Implen, Westlake Village, CA). Following RNA isolation and quantification, samples were normalized to a concentration of 1 µg/µl. Total RNA (200 ng) from samples were reverse-transcribed using the iScript Reverse Transcription Supermix for RT-qPCR (Bio-Rad, Hercules, CA) following the manufacturer’s protocol.

### qPCR Analysis

qPCR analysis was done using the primers listed in Table 1, in an Eppendorf MasterCycler RealPlex (ThermoFisher Scientific, Waltham, MA) following the standard protocol for forty cycles (denature at 95°C for 10 s, annealing at primer TM for 30s and elongation at 72°C for 15 s ×40), with postamplification melt curve analysis. Ribosomal protein (RPL32) and Glyceraldehyde 3-phosphate dehydrogenase (GAPDH) were used as reference (control) genes (Scharlaken et al. 2008) for standard quantification purposes. Primer efficiency was determined using the standard curve analysis method (Larionov et al. 2005). To determine primer efficiency we took 1 µl of each cDNA sample, those samples were pooled and serially diluted in five points at 1:10 dilutions. Samples were run in a qPCR using the established protocol. Primer efficiency % was calculated using the efficiency() command from the ‘qpcR’ package (v. 1.4-1) in the program R. qPCR reactions were prepared with 1 µl of cDNA as a template in a master mix of 1 µl of primers at [10 nM], 5 µl of iTaq Universal SYBR Green Supermix (Bio-Rad), and 3 µl of water for a final volume of 10 µl. cDNA from brain tissue was used to measure JHamt, ILP-1, ILP-2, IRS, and TOR, while cDNA from abdomen tissue was used to measure Vg (Nilsen et al. 2011; Doke 2017; Bresnahan et al. 2022). Gene expression was calculated using the geometric mean analysis method described by Vandesompele et al. (2002). The relative expression values presented are relative to the control group.

### Statistics

Data normality was analyzed with a Shapiro–Wilk test. After confirming normality of the data and test assumptions, to determine whether gene expression was significantly different between treatment groups within age, we performed a unpaired *t*-test with a Welch correction for the 1 d olds to compare control (−/−) and antibiotic treated (+/+). An ANOVA with a Welch correction was used for the 7- and 14-d-old bees to compare gene expression among the four treatment groups. A Tukey test was used as a post hoc test in a pairwise multiple comparison.

A principal component analysis (PCA) was performed to verify if the expression patterns demonstrated any clustering given the different antibiotic treatments. PCA was run in a singular value decomposition (SVD) method with imputations used to calculate principal components with a probability of 95%. To further visualize and assess the gene expression patterns among age and treatment, we examined gene expression in a heatmap by average-linkage hierarchical clustering based on Euclidean distance (Rajalingam and Ranjini 2011). Data were analyzed using the statistical program R (RStudio, Inc.) v. 3.5.2 (2018-12-20) and the package pheatmap (v. 1.0.12). Graphs and figures were done Graph Pad Prism 6.0 (GraphPad software, La Jolla, CA) and RStudio package Mass (v. 7.3-53).

## Results

**Fig. 1.**
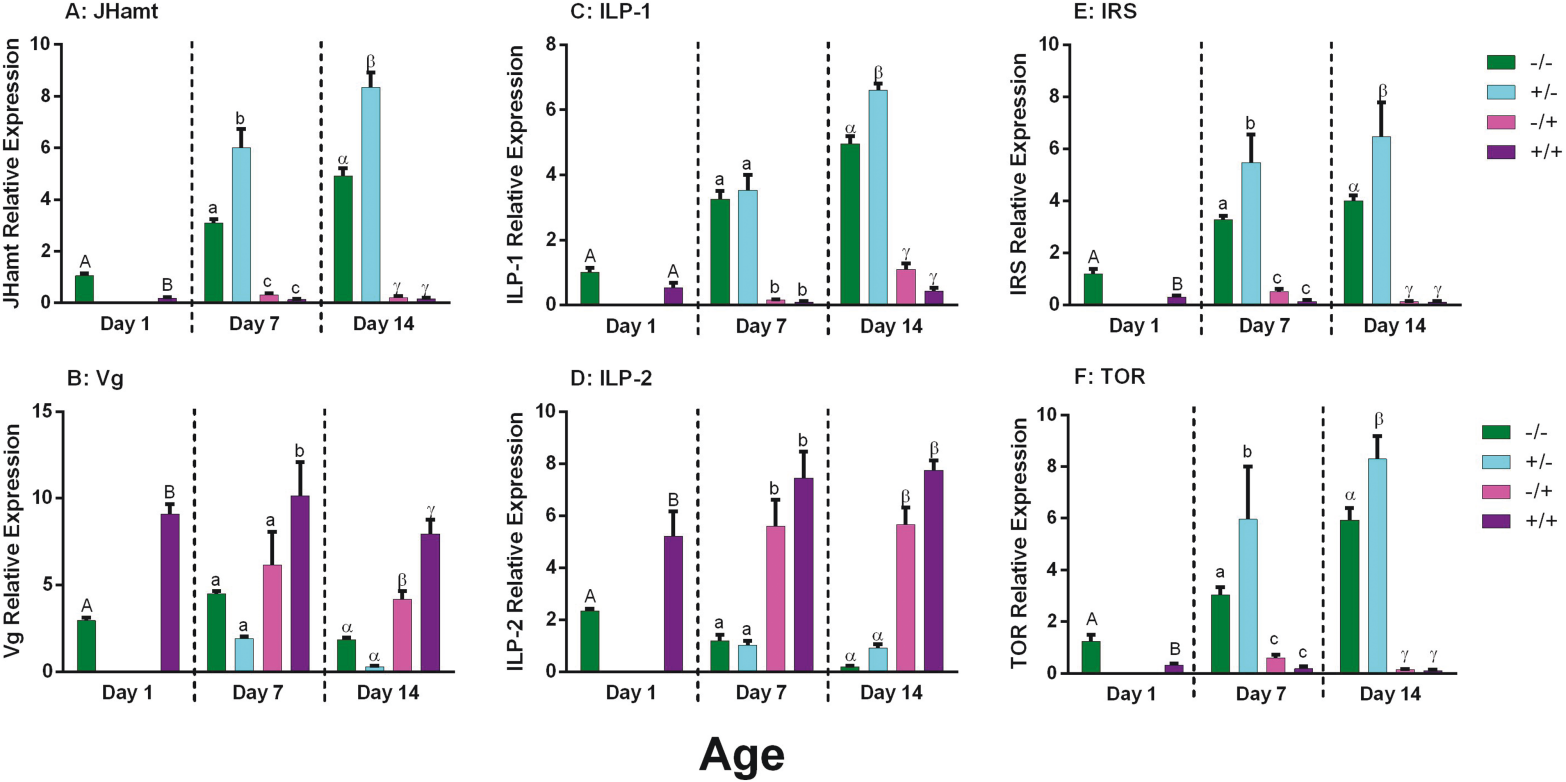
Expression pattern of development-associated genes. These results demonstrate different gene expression pattern by treatment and age. Green = control bees (−/−), blue = development exposed bees (+/−), magenta = adult exposed bees (−/+), and purple = developmental and adult exposed bees (+/+). Relative expression was measured using the geometric mean analysis method. Capital letters show differences between treatments groups at 1 d of age, noncapitalized letters show differences between treatments groups at 7 d of age, and Greek letters show differences between treatments groups at 14 d of age. *N* = 10 for each treatment group by age. Samples: day 1 *n* = 20, day 7 *n* = 40, day 14 *n* = 40, total *N* = 100. Data reported by mean ± SEM of gene relative expression.

Antibiotic treatments had an effect on the typical expression pattern of regulatory genes. In control bees, expression profiles appeared to follow typical patterns: JHamt, ILP-1, IRS, and TOR expression increase over adult development time, while Vg and ILP-2 decrease over time (Fig. 1). Bees of 1 d of age show significant difference in gene expression for most genes between the two groups. Antibiotic-treated bees (+/+) had a higher gene expression of Vg and ILP-2 (Vg: *t* = 10.76, df = 18, *P* < 0.000; ILP-2: *t* = 4.02, df = 18, *P* < 0.0001) and a lower gene expression of JHamt, IRS, and TOR when compared with the control (JHamt: *t* = 7.96, df = 18, *P* < 0.0001; IRS: *t* = 4.16, df = 18, *P* < 0.0001; TOR: *t* = 3.29, df = 18, *P* = 0.004). No significant differences was observed for ILP-1 gene expression (*t* = 1.83, df = 18, *P* = 0.08).

At 7 d of age gene expression for all genes was found to be significantly different across the treatment groups (JHamt: *F*_(3,36)_ = 49.50, *P* < 0.0001; Vg;: *F*_(3,36)_ = 18.64, *P* < 0.0001; ILP-1: *F*_(3,36)_ = 36.41, *P* < 0.0001; ILP-2: *F*_(3,36)_ = 49.50, *P* < 0.0001; IRS: *F*_(3,36)_ = 19.67, *P* < 0.0001; TOR: *F*_(3,36)_ = 6.47, *P* = 0.001). Post hoc comparisons indicate that bees in antibiotic-treated colonies (−/+ and +/+) at this age, had a higher gene expression of Vg and ILP-2 and a lower gene expression on JHamt, ILP-1, IRS and TOR than the −/− and +/− groups. Moreover the +/− group showed a higher gene expression on JHamt, IRS, and TOR when compared with the control (−/−) group (mean diff: −4.75, −4.19, and −4.32, respectively). Similar differences of gene expression were found in bees of fourteen days old, where −/+ and +/+ bees had a higher gene expression of Vg and ILP-1 and a lower expression of JHamt, ILP-1, IRS, and TOR when compared with the −/− and +/− groups (JHamt: *F*_(3,36)_ = 157.7, *P* < 0.0001; Vg; *F*_(3,36)_ = 60.90, *P* < 0.0001; ILP-1: *F*_(3,36)_ = 10.29, *P* < 0.0001; ILP-2: *F*_(3,36)_ = 62.37, *P* < 0.0001; IRS: *F*_(3,36)_ = 20.54, *P* < 0.0001; TOR: *F*_(3,36)_ = 72.12, *P* = 0.001). The +/− also showed a higher gene expression of JHamt, ILP-1, IRS, and TOR than the −/− group at this age (mean diff: −7.15, −3.48, −5.23, and −7.05, respectively).

**Fig. 2.**
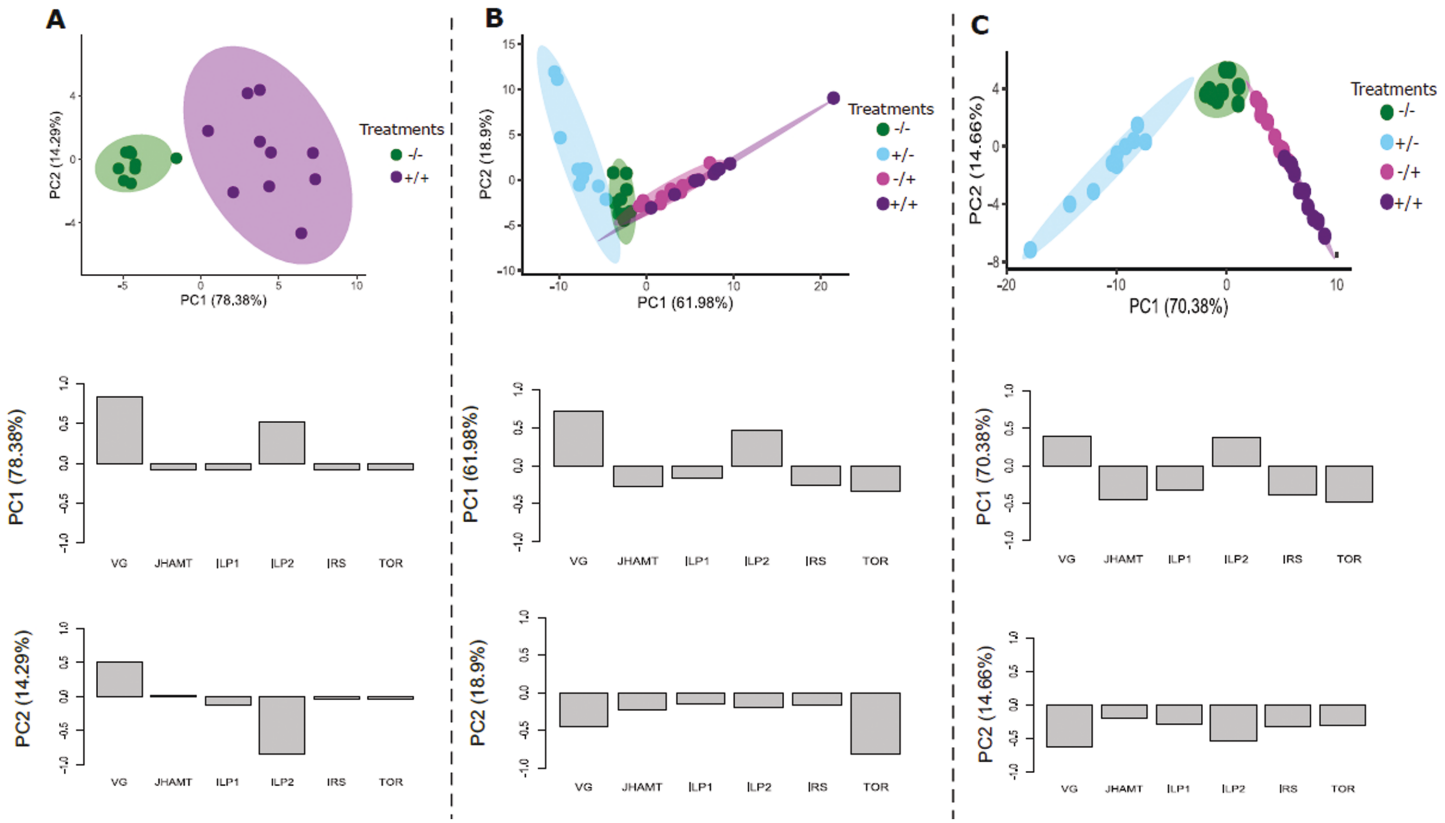
Principal component analysis (PCA) of gene expression due to treatment and age. Green circles = control bees (−/−), blue circles = development exposed bees (+/−), magenta circles = adult exposed bees (−/+), and purple circles = developmental and adult exposed bees (+/+). (A) One-day-old bees. X and Y axes show principal component 1 and principal component 2 that explain 78.38 and 14.29% of the total variance, respectively. (B) Seven-day-old bees. X and Y axes show principal component 1 and principal component 2 that explain 61.98 and 18.9% of the total variance, respectively. (C) Fourteen-day-old bees. X and Y axis show principal component 1 and principal component 2 that explain 70.38% and 14.66% of the total variance, respectively. Prediction ellipses are such that with probability 0.95. Bar plots show the most contributive loadings (genes) to the value in the PCA. Samples: day 1 *n* = 20, day 7 *n* = 40, day 14 *n* = 40, total *N* = 100.

PCA by age showed clustering based on gene expression. In bees of 1 d of age, gene expression clusters into two groups, those not treated and treated with antibiotics (Fig. 2A). On 7 d of age (Fig. 2B), three different clusters were observed for gene expression, where expression of −/+ and +/+ form one cluster and +/− and −/− form two different clusters. In 14 d of age (Fig. 2C), a similar pattern as the one showed at 7 d of age is seen, with the difference of the −/+ group indicated by greater separation from the +/+ group.

**Fig. 3.**
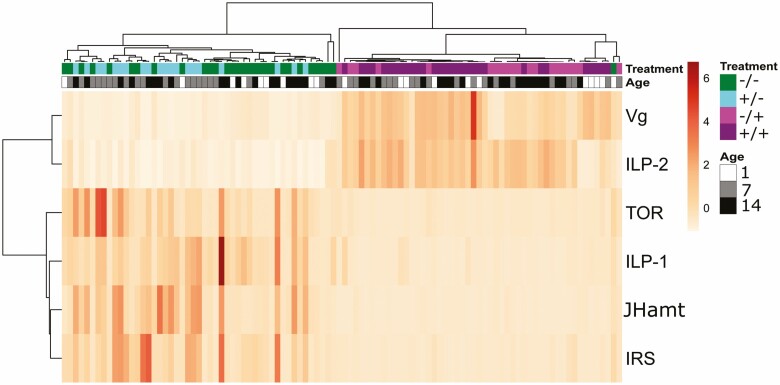
Gene expression heat map of development-associated genes. Rows are centered; unit variance scaling is applied to rows. Both rows and columns are clustered with average-linkage hierarchical clustering based on Euclidean distance. Six rows (genes), 100 columns. Green = control bees (−/−), blue = development exposed bees (+/−), magenta = adult exposed bees (−/+), and purple = developmental and adult exposed bees (+/+). White = 1-d-old bees, gray = 7-d-old bees, and black = 14-d-old bees. Heat map is based on average of relative expression among the individuals per group on each gene in a scale (orange to red gradient) lowest expression (−2, orange) to highest expression (7, red).

When we assessed gene expression patterns among age and treatment in a heatmap, we identified that gene expression clustered by antibiotic treatments (Fig. 3). Expression of ILP-1, JHamt, IRS, and TOR form a cluster while Vg and ILP-2 expression forms a second cluster. Examining each of those clusters shows that higher Vg and ILP-2 expression is not only related to younger bees in the control group but also related to the −/+ and +/+ groups. An inverted pattern is seen for the ILP-1, JHamt, IRS, and TOR gene cluster where a higher expression is observed for older bees in the control group. Higher expression of forager-like profiles is observed earlier in the +/− group, from age 7–14. Throughout the experiment, +/+ expression profiles resemble that of young workers.

## Discussion

The principal inference based on gene expression changes is that antibiotic treatment alters typical behavioral development through coordinated changes in metabolic and neuroendocrine regulation supporting the *regulatory effect hypothesis*. The timing of antibiotic treatment in adult stage (+/+ and −/+) versus in juvenile stage (+/−) altered gene expression associated with typical nutritional and behavioral profiles.

In gene expression profile and behavioral development, in comparison to control bees (−/−) not exposed to antibiotics, the +/+ and −/+ bees, that were exposed to antibiotics as adults, reflected a delayed development pattern with low JHamt, ILP1, IRS, and TOR versus high Vg and ILP2 expression. The delayed development included continued performance of cleaning and nursing jobs even at 14 days of age (Ortiz-Alvarado et al. 2020).

In contrast the +/− group bees, that were only exposed to antibiotics during early or larval development showed a precocious development pattern with high JHamt, ILP1, IRS, and TOR expression, and low Vg and ILP2 expression (see panels of Fig. 1). The precocious development included onset of foraging tasks at 7–10 d of age for 18% of the individuals in the +/− group (Ortiz-Alvarado et al. 2020). This is ca. 2 wk earlier than typical onset of foraging in bees (Seeley 1982).

The gene expression pattern of bees demonstrating precocious development is similar to pattern observed in bees switching from nursing tasks to foraging. Increase in ILP-1 production is thought to influence foraging behavior by action on neuroendocrine pathways affecting peripheral cells such as the fat bodies in the abdomen (Nilsen et al. 2011). The increase of IIS pathway via ILP-1 is related to lipid loss and leads to faster behavioral development, whereas increased ILP-2 relates to nurse-like profiles. In contrast, the pattern observed in delayed development is consistent with gene expression that is indicative of suppression of reduced transduction of the IIS pathway and suppressed JH synthesis, a profile known to be related to high nutrition profiles as those of nurses (Ament et al. 2008; Nilsen et al. 2011, Ihle et al. 2014).

IRS and TOR are necessary for JH content in bees. Studies with –IRS and –TOR knockdowns produced bees with lower amounts of JH (Mutti et al. 2011), demonstrating their importance in metabolic pathways and key elements in behavioral development. Similarly, TOR pathway is upstream of Vg synthesis (Patel et al. 2007). Regulation of growth by TOR acts through the IIS pathway (Wheeler et al. 2014), and an upregulation of TOR could further explain the downregulation of Vg observed in bees as they age and change tasks, with TOR acting as an agonist, similar to ILP-2 and downregulation of the IIS pathway.

Incidentally, our results show the immature environment can influence adult development. Treating honey bee larvae with antibiotics produced adult bees that began foraging early when placed in a control colony and late if treatment continued. In both treatments, these bees emerged with higher lipid amounts than the nontreated bees (Ortiz-Alvarado et al. 2020), suggesting a change in the nutritional metabolism during development triggered by antibiotics. This is consistent with the idea that the bees have reached a critical ‘check-point’ in adiposity, leading to alternate adult behavioral development in the absence of further antibiotic treatment. This is similar to observation in *Drosophila melanogaster* (Loew, 1862 [Diptera: Drosophilidae]), where the stage of adult development and maturation is reached by surpassing a critical weight ‘check point’, which is regulated by genetic mechanisms that coordinate this development progression by nutritional intake (Britton et al. 2002; Ikeya et al. 2002).

The observation that bees exposed for different duration and stage of development (−/+ and +/+) to have similar behavior and gene expression, namely delayed development, refutes the *toxic effect hypothesis*. Changes in behavior cannot be a direct consequence of toxicity to the bee due to prolonged exposure to antibiotics, instead the Regulatory Effects Hypothesis is supported.

We further hypothesize the microbiota to be an important factor in the timing of behavioral development in honey bees, as its composition changes with antibiotics (Thompson et al. 2017; Zheng et al. 2017). The connection between brain gene expression and abdomen adiposity points to regulation via the gut–brain axis (Forsythe and Kunze 2013; Mayer et al. 2015). In the bee brain and abdomen tissues are linked through the IIS pathway that promotes a stable lipid loss typically promoting foraging behavior (Ament et al. 2011, Nilsen et al. 2011). The IIS system feeds into hormonal signaling timing the development and maturation of an organism (Britton et al. 2002; Ikeya et al. 2002; Nijhout and McKenna, 2018). Recent studies have shown the gut microbiota may also influence behavioral maturation by regulating hormonal signaling important to development and metabolism (Martinson et al. 2012; Moran et al. 2012; Rayamann et al. 2017; Zheng et al. 2017; Jones et al. 2018; Ortiz-Alvarado 2019).

We infer antibiotics, through their effect on the microbiota composition, disrupt or alter the metabolic regulatory network. The +/− group bees developed even faster and expressed even at higher level foraging related genes in comparison to untreated individuals (−/−). The interpretation is that likely, the +/− bees were at a set point (or critical “check-point”) conducive to begin lipid loss and foraging related changes (Toth et al. 2005). Upon regaining microbiota important for metabolism (e.g. *Gilliamella apiciola* (Kwong and Moran, 2013 [Orbales: Orbaceae]); Zheng et al. 2016; Bonilla-Rosso and Engel 2018) through nest-mates interactions after being placed in an antibiotic-free colony, this interaction may conduct rapid lipid loss and accelerated rate of development that reflects on gene expression. This interpretation also explains the observation on −/+ and +/+ group bees. These groups were exposed to antibiotics during adulthood and without this microbial signaling the lipid metabolism and behavioral gene expression changes would be delayed in both group of bees.

Here we describe distinct patterns in gene expression related to the timing of antibiotic exposure by examining target genes in a tissue-specific manner. In the future, to demonstrate the link between antibiotic treatment and microbiota change in the same individuals, microbiota composition should also be determined, preferably through microbiome sequencing (Bobay et al. 2020; Vernier et al. 2020). In addition, a control for tissue specificity of gene expression may require examination of expression of all genes in all target tissues. Ultimately, the combination of genic patterns in adipose tissue, brain, and microbiota composition may underlie the phenotypic display of worker behavioral development.

In conclusion, our results demonstrate that the timing of exposure to antibiotics alters gene expression associated with behavioral development. Antibiotic effects on individual behavior highlight need for further focus on the gut–brain axis and microbiota role on honey bee social organization.
